# Mapping QTL influencing gastrointestinal nematode burden in Dutch Holstein-Friesian dairy cattle

**DOI:** 10.1186/1471-2164-10-96

**Published:** 2009-03-02

**Authors:** Wouter Coppieters, Ted HM Mes, Tom Druet, Frédéric Farnir, Nico Tamma, Chris Schrooten, Albert WCA Cornelissen, Michel Georges, Harm W Ploeger

**Affiliations:** 1 Unit of Animal Genomics, Faculty of Veterinary Medicine and Centre for Biomedical Integrative Genoproteomics, University of Liège (B43), Liège, Belgium; 2 Department of Infectious Diseases and Immunology, Faculty of Veterinary Medicine, Utrecht University, Utrecht, The Netherlands; 3 Holland Genetics, Arnhem, The Netherlands

## Abstract

**Background:**

Parasitic gastroenteritis caused by nematodes is only second to mastitis in terms of health costs to dairy farmers in developed countries. Sustainable control strategies complementing anthelmintics are desired, including selective breeding for enhanced resistance.

**Results and Conclusion:**

To quantify and characterize the genetic contribution to variation in resistance to gastro-intestinal parasites, we measured the heritability of faecal egg and larval counts in the Dutch Holstein-Friesian dairy cattle population. The heritability of faecal egg counts ranged from 7 to 21% and was generally higher than for larval counts. We performed a whole genome scan in 12 paternal half-daughter groups for a total of 768 cows, corresponding to the ~10% most and least infected daughters within each family (selective genotyping). Two genome-wide significant QTL were identified in an across-family analysis, respectively on chromosomes 9 and 19, coinciding with previous findings in orthologous chromosomal regions in sheep. We identified six more suggestive QTL by within-family analysis. An additional 73 informative SNPs were genotyped on chromosome 19 and the ensuing high density map used in a variance component approach to simultaneously exploit linkage and linkage disequilibrium in an initial inconclusive attempt to refine the QTL map position.

## Background

Parasitic gastroenteritis (PGE) caused by trichostrongylids and strongylids remains an important issue for cattle husbandry world-wide including in developed countries. Treatment and prophylaxis relies to a large extent on the use of broad spectrum anthelmintics combined with proper management practices. Although these measures are rather effective, nematode infestation is only second to mastitis in terms of health costs to dairy farmers, estimated at 90 million € annually for the Netherlands only. These costs result not only from the use of anthelmintics but also from the production losses due to sub-clinical infestation (e.g. [1]).

Despite their efficacy, the systematic and extensive use of anthelmintics causes concerns with regards to (i) the negative effect on the development of natural immunity, (ii) consumer concerns regarding drug residues in food products and the environment, and (iii) the increasing incidence of parasite resistance against available anthelmintics. Complementary control strategies are thus desirable and a number of approaches are being explored including the use of nematophagous fungi, tannins, immunonutrition, vaccination (e.g. [2,3]), as well as selective breeding for enhanced resistance or resilience (e.g. [4]).

Evidence for an inherited component in susceptibility to gastrointestinal nematodes in ruminants stems from (i) the observation of differences in susceptibility within and between breeds – particularly in small ruminants [5-11] -, (ii) the response to divergent selection [12,13], as well as (iii) estimates of heritability – ranging from 0.1 to 0.8 – measured within breeds [14,13-21]. These observations have spurred efforts to increase innate resistance by selective breeding (e.g. [4]) as well as to identify the genes and QTL that underlie this genetic variation (e.g. [22,23]). In sheep, significant associations have been reported for the MHC (e.g [24,25]) and IFNγ (e.g. [26]) loci, while three genome scans have resulted in few significant but many suggestive [27-29]. Ongoing efforts to map QTL influencing resistance to gastrointestinal parasites in an Angus population have been described, but – to the best of our knowledge – no QTL locations have yet been reported (e.g. [13]). Positional cloning experiments in livestock are advantageously complemented by similar approaches conducted in rodent models (e.g. [30]). In humans, a whole genome linkage scan performed in an isolated Nepalese population identified two loci with unequivocal effect on susceptibility to *Ascaris *infection (e.g. [31]).

Identifying QTL and gene variants influencing parasite burden paves the way towards marker assisted selection (MAS) for increased resistance. MAS may be particularly effective for this trait as it has relatively modest heritability and is tedious to measure. Parasite burden is typically "overdispersed" with most animals being virtually devoid of parasites and a few being heavily infected and responsible for most of the infestation pressure. Identifying such "shedders" (contributing most to pasture contamination) based on their inherited predisposition, might be an effective way to reduce overall parasite transmission.

We herein describe a whole genome scan to map QTL influencing gastro-intestinal nematode burden in a Holstein-Friesian "daughter design" [32] in which we exploit selective genotyping (e.g. [33]). We subsequently combine linkage and linkage disequilibrium mapping (e.g. [34-36]) in an attempt to refine the map position of one of the QTL identified in the genome scan.

## Results

### Estimating the heritability of gastrointestinal nematode burden in the Dutch Holstein-Friesian dairy cattle population

**Figure 1:**
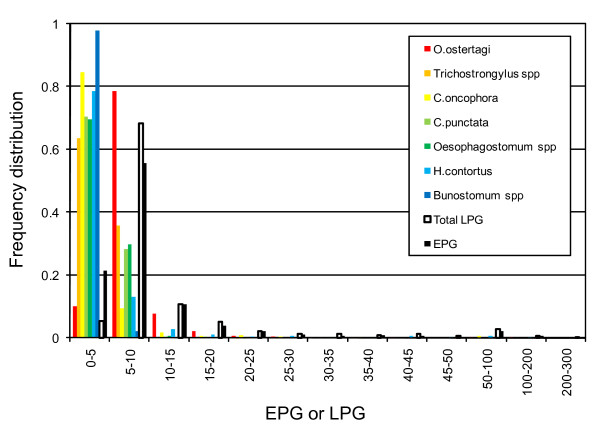
**Frequency distribution of the number of genus/species-specific larvae per gram of faeces (coloured bars) and number of nematode eggs per gram of faeces (EPG, black bar) in a sample of 1,419 Dutch Holstein-Friesian dairy cows**. "Total LPG" (white bar) corresponds to the overall sum of the genus/species-specific LPG.

To estimate the heritability of gastrointestinal nematode burden in Dutch dairy cattle, we collected faeces from 1,420 cows between June and August 2000. Selected animals were between two and six years of age, more than two months away from the last and next calving date to avoid variation associated with peri-parturient relaxation in immunity, and grazing as infestation is known to be pasture-borne [37,38]. They originated from 605 herds. Nematode eggs per gram of faeces (EPG) were counted as described [39]. To estimate the parasite burden by species, we performed coprocultures and counted the number of genus or species-specific (*Bunostomum *spp, *Cooperia oncophora, Cooperia punctata, Haemonchus contortus, Oesophagostomum *spp, *Ostertagia ostertagi, Trichostrongylus *spp) larvae per gram of faeces (LPG) as described [40]. Fig. 1 shows the frequency distribution of EPG and LPG in this data set.

To measure the heritability of nematode burden, we transformed EPG and LPG in LEPG (=Log_10_(EPG+1)) and LLPG (=Log_10_(LPG+1)) respectively. We then estimated narrow-sense heritabilities (*h*^2^) using a mixed model including an overall mean, a fixed parity effect, an individual animal effect, a random herd effect and a random error (e.g. [41]). Genealogical information, including data on 3190 ancestors up to 14 generations from present, was obtained from HG (Arnhem, The Netherlands) and used to compute the additive relationship matrix. Variance components were estimated using AI-REML [42]. Table 1 summarizes the obtained results. LEPG and LLPG decreased significantly with parity as expected (LEPG: parity 1: -0.06; 2: -0.13; 3: -0.24; 4: -0.21; p = 2.1 × 10^-5^; LLPG: parity 1: -0.19; 2: -0.23; 3: -0.36; 4: -0.37; p = 1.5 × 10^-6^). In general, the heritability for faecal egg counts (LEPG) was higher (21%) than for the larval phenotypes (0–12%), with noticeable exception for *Haemonchus *larval counts (25%). Because EPG had a relatively high heritability and as it is also the simplest phenotype to measure, it was the only one selected for further analysis.

**Table 1:** Variance components of gastrointestinal nematode burden in Dutch Holstein-Friesian dairy cattle.

	**Phenotype**								
**Random effect**	**LEPG**	**LLPG**							
		***Bun.***	***C.onc.***	***C.pun.***	***Hae.***	***Oes.***	***Ost.***	***Tric.***	**TOT**
Animal ($\sigma_{A}^{2}$/$\sigma_{P}^{2}$) (Stand.error)	0.21 (0.05)	0.00 (0.02)	0.07 (0.04)	0.05 (0.03)	0.25 (0.05)	0.03 (0.03)	0.06 (0.03)	0.00 (0.02)	0.12 (0.04)
Herd ($\sigma_{H}^{2}$/$\sigma_{P}^{2}$) (Stand.error)	0.21 (0.02)	0.12 (0.03)	0.05 (0.02)	0.07 (0.03)	0.34 (0.02)	0.28 (0.03)	0.05 (0.02)	0.10 (0.02)	0.12 (0.02)
$\sigma_{P}^{2}$	0.212	0.000	0.103	0.026	0.137	0.017	0.105	0.015	0.226

$\sigma_{P}^{2}$: phenotypic variance; $\sigma_{A}^{2}$: additive genetic variance; $\sigma_{H}^{2}$: variance due to the herd effect.

LEPG: log_10_(EPG+1) where EPG = eggs per gram of faeces; LLPG: log_10_(LPG+1) where LPG = number of larvae per gram of faeces. Bun.: *Bunostomum *sp.; C.onc.: *Cooperia oncophora*; C.punc.: *Cooperia punctata*; Hae.: *Haemonchus *sp.; Oes.: *Oesophagostomum *sp.; Ost.: *Ostertagia *sp.; Tri.: *Trichostrongylu*s sp.; TOT: sum over all species.

### A whole genome scan using selective genotyping identifies two QTL influencing nematode burden in dairy cattle

**Figure 2:**
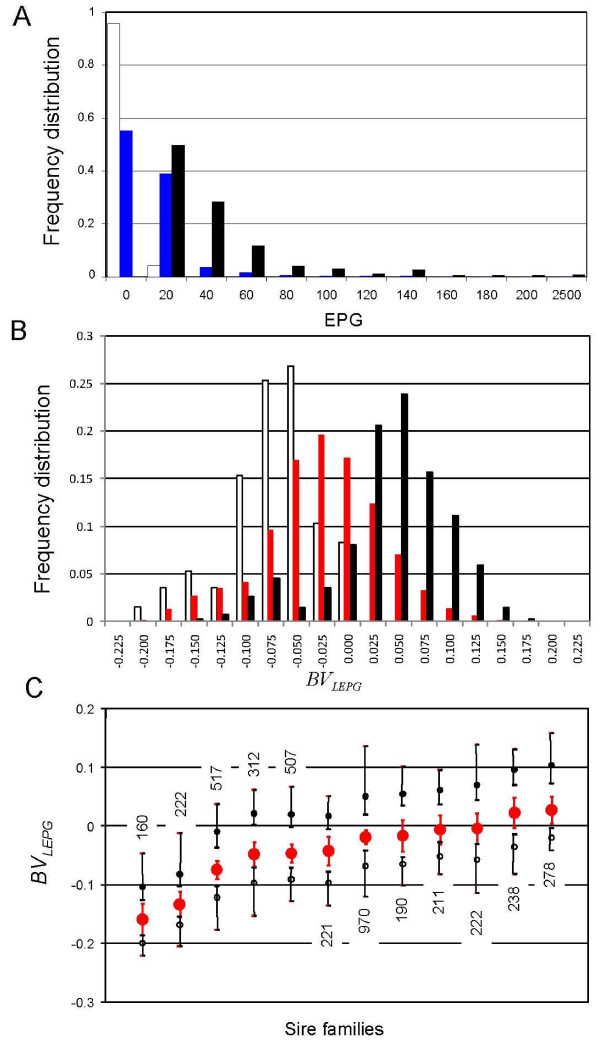
**Frequency distribution of the number of nematode eggs per gram of faeces (EPG) (A), as well as of the breeding value for Log_10_(EPG+1) (BV_LEPG_) (B), for 4,048 Holstein-Friesian dairy cows belonging to twelve paternal half-sib pedigrees (EPG: blue; BV_LEPG_: red)), as well as for the daughters selected for the QTL scan (white: lower tail, black: upper tail)**. **(C) **Mean BV_LEPG _± 1.96 SE (red circle + error bars), as well as mean (circle) and range (error bars) of the upper (black) and lower (white) tails for each of the twelve daughter group. The number of daughters before selection is shown for each sire.

To map QTL influencing nematode burden in dairy cattle, we collected faeces and venous blood from 4,053 Dutch Holstein-Friesian dairy cows between July and September 2002. The cows were selected on age (2^nd^, 3^rd ^or 4^th ^calving between November 2001 and March 2002), sire (one of twelve sires) and herd (533 herds). Numbers of nematode eggs per gram of faeces (EPG) were determined for each animal using the rapid variant of a high-throughput isolation procedure described in Mes et al. [39], which is described in more detail in the Materials and Methods. Resulting EPG were log-transformed (LEPG), and breeding values (BV_LEPG_) computed for each cow using the same mixed model as in the previous analysis augmented with a regression on the number of "days in milk" (e.g[41]). Variance components were estimated using AI-REML [42]. LEPG decreased very significantly with increasing parity (parity 2: 0.12; 3: 0.04; 4: 0.00; p = 1.7 × 10^-11^), and by 0.0006 per day in milk (p = 0.003). The heritability of LEPG in this data set was only 0.074 (standard error 0.027), thus considerably lower than the previous estimate. The herd effect accounted for 22.6% of the variance (standard error 0.013). Half-sib ANOVA analysis of LEPG using a sire model highlighted the extreme significance of the sire effect (p < 4 × 10^-14^), but led to a comparably low *h*^2 ^estimate of 0.09 assuming that the correlation between half-sibs ("intraclass correlation") equals *h*^2^/4 (e.g. [41]). Fig. 2 shows the distribution of EPG and BV_LEPG _values across the entire data set, as well as the mean and range of BV_LEPG _values for each of the twelve sire families. As EPG were measured using distinct procedures in the 2000 and 2002 campaigns, we did not perform a joint analysis of both data sets.

**Figure 3:**
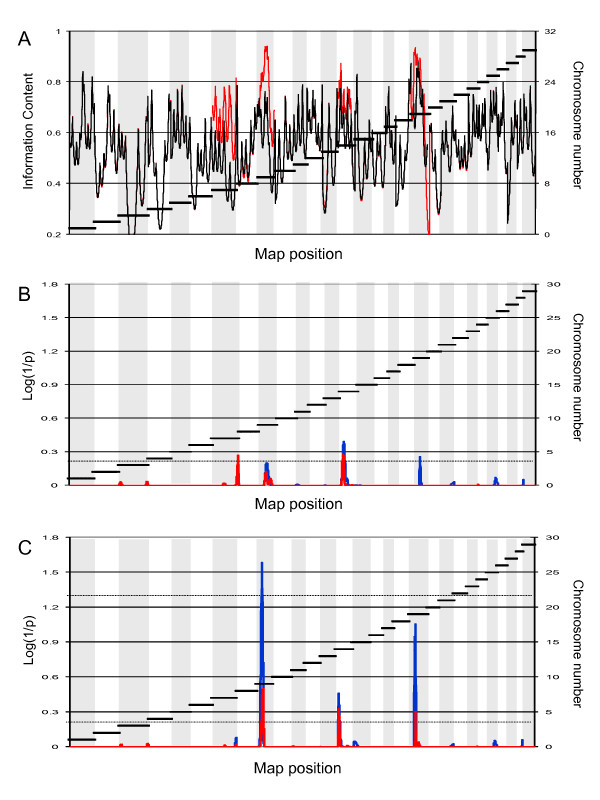
**(A) Information Content of the map used for the initial genome scan (153 autosomal microsatellites) (black), and after increasing the marker density on chromosomes 7, 9, 14 and 19 (red)**. **(B) **Location scores (log(1/p)) obtained along the bovine genome in the initial pass for BV_LEPG _(red) and EPG (blue). The suggestive threshold is shown as a horizontal dotted line. **(C) **Location scores (log(1/p)) obtained along the bovine genome in the second pass for BV_LEPG _(red) and EPG (blue). The threshold (p < 0.05) for genome-wide significance is shown as a horizontal dotted line.

Within each sire family, we selected the top and bottom ~10% of the daughters on BV_LEPG _for a total of 768 (2 × 384) cows (Fig. 2C). Selected daughters and their sires were genotyped for a panel of 153 microsatellite markers spanning the bovine autosomes. The markers were chosen based on their chromosomal location and heterozygosity in the 12 sires. We constructed marker maps using CRIMAP [43]. Marker order was in perfect agreement with published maps (e.g. [44]). The maps spanned a combined 28.79 Morgan (Kosambi), in good agreement with the 31.59 Morgan of the Ihara et al. [44] male map length. The average distance between adjacent markers was thus 22.85 cM (Kosambi). Fig. 3A shows the information content [45] of the corresponding marker map.

QTL analysis was performed using a previously described non parametric rank-based method for outbred half-sib pedigrees that is particularly suited for phenotypes that are not normally distributed including parasite counts [45]. Significance thresholds were determined by permutation [46]. QTL analysis was performed both for BV_LEPG _and for EPG. Fig. 3B shows the results obtained by across-family analysis in this first pass genome scan. The suggestive threshold (log_10_(1/*p*) = 0.2), i.e. the threshold expected to occur on average once per genome scan under the null hypothesis [47], was exceeded on four chromosomes: BTA7, 9, 14 and 19.

**Figure 4:**
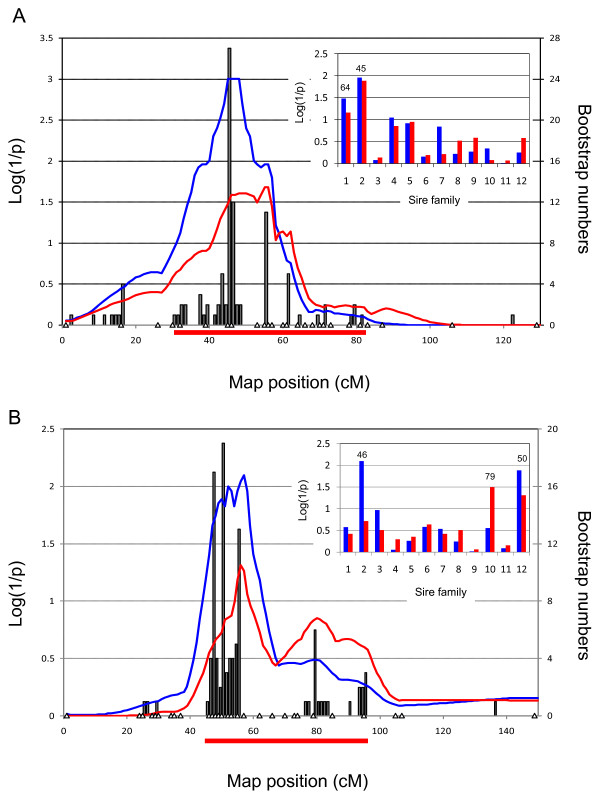
**Chromosome-wide location scores (log(1/p)) along the chromosome 9 (A) and 19 (B) maps, for BV_LEPG _(red) and EPG (blue)**. Marker positions are marked by white triangles. The distribution of most likely QTL position across 1,000 bootstrap samples are marked by gray bars; the resulting 95% confidence interval is shown as a red horizontal line. The insets shows the results of the within family analysis for BV_LEPG _(red) and EPG (blue). The most likely QTL position (cM) is given above the bar graphs for families exceeding chromosome-wide significance.

For these chromosomes, we increased the microsatellite density from six to 17 (BTA7), seven to 26 (BTA9), six to 18 (BTA14) and seven to 29 (BTA19). Maps were re-constructed using CRIMAP and their information content shown to increase by 23% on average (Fig. 3A). We repeated the whole genome QTL scan including the genome-wide permutations test. While the statistical evidence supporting the QTL did not increase for chromosomes 7 and 14, on the contrary, the signal intensity increased quite dramatically for chromosomes 9 and 19, reaching genome-wide significance for BTA9 (p = 0.025) and near genome-wide significance for BTA19 (p = 0.08)(Fig. 3C). Note that the associated nominal p-values are respectively 6 × 10^-4 ^and 1.8 × 10^-3^. Fig. 4A and 4B show the detailed location scores obtained on BTA9 and BTA19 including the confidence intervals for the QTL location determined by bootstrapping [48,49]. Within family analysis indicates that at least two of the 12 sires are likely to be heterozygous for the BTA9 QTL and three for the BTA19 QTL (Fig. 4A and 4B).

Further examination of the within-family results across the entire genome revealed an additional six chromosomal locations with genome-wide suggestive evidence for QTL influencing nematode burden (Table 2). Note that the corresponding threshold does not account for the analysis of multiple (albeit correlated) phenotypes (two) and multiple families (12 families).

**Table 2:** Genome-wide suggestive QTL identified only by within-family analysis.

BTA (OAR*)	**Most significant position or interval**	**Family**	**Trait**	**Log(1/p)**
11 (3)	BP38-BM7169	2	EPG	1.54
14 (9)	BMS1678	12	BV_LEPG_	0.80
21 (18)	TGLA122-BMS743	11	BV_LEPG_	1.00
21 (18)	BMS743-CSSM26	12	EPG	0.73
24 (23)	BMS1743-TGLA435	10	BV_LEPG_	0.87
25 (24)	BM737-BMS1353	4	EPG/BV_LEPG_	0.87/1.25
27 (26)	BM3507-INRA183	5	BV_LEPG_	0.96

**OAR: **Sheep chromosome numbers have been added to facilitate cross-referencing with published genome scans in this species.

### Attempting to refine the map position of the BTA19 QTL using combined linkage and linkage disequilibrium (LD) analysis

**Figure 5:**
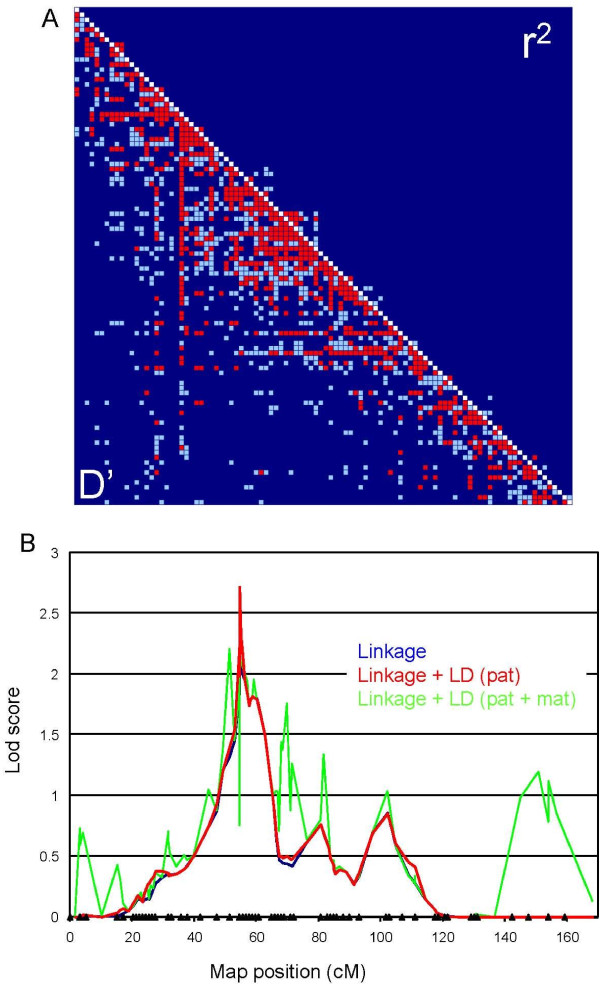
**(A) Pairwise LD between BTA19 markers measured as *D' *(lower left) and as *r*^2 ^(upper right)**. Values above 0.3 are highlighted in red, values between 0.2 and 0.3 in light blue, and values < 0.2 in dark blue. **(B) **Results of the combined linkage plus LD analysis for LEPG. The triangles mark the map position (cM) of microsatellites and SNPs added for fine-mapping. The location score profile in blue correspond to the linkage signal, in red to the linkage plus the LD signal from the paternal chromosomes, and in green to the linkage plus de complete LD signal. The Y-axis corresponds to lod scores.

To refine the map position of the BTA19 QTL, two SNP-lex^R ^sets (Applied Biosystems, Foster City) were used to genotype the 12 sires and their selected daughters for an additional 96 SNPs. The corresponding BTA19 SNPs were selected from the available public domain SNPs  on the basis of their predicted chromosomal location, detection in Holstein-Friesian sequence traces, and prior information about minor allele frequency (MAF). 73 of the 96 SNPs proved to be polymorphic in our pedigree material. The distribution of MAFs in the Dutch Holstein-Friesian population is shown in the additional file 1. The 73 polymorphic SNPs were included in the BTA19 map using CRIMAP [43]. Markers that could not be positioned by linkage analysis were placed according to their location on the bovine genomic sequence (Build 4.0). The obtained marker map is shown in Fig. 5B with additional details provided in additional file 2. It comprises a total of 101 markers with an average distance between adjacent markers of 1.7 cM. We measured the level of LD for all marker pairs using both D' and r^2^. The resulting LD map is shown in Fig. 5A.

This higher density BTA19 map was used in a variance component analysis that simultaneously extracts information from linkage as well as LD (e.g. [34-36]). The phenotype considered was LEPG, corrected for herd effect, parity effect and effect of days in milk as estimated from the complete data set (4,053 cows). The corresponding mixed model included an individual genotype effect, an individual animal effect, and a random error. The obtained results are shown in Fig. 5B.

Exploiting only the linkage signal provided by the paternal chromosomes (i.e. considering that the 24 paternal chromosomes and 768 maternal chromosomes are unrelated, and letting the covariance between the genotype effects of half-sisters only depend on the sharing of paternal chromosomes) in essence recapitulates the results obtained with the rank-based QTL mapping using the lower density marker map (Fig. 4B). The most likely position of the QTL is at position 54.48 cM (z = 2.1) coinciding with the URB026-TGLMA51 interval. Extracting LD information from the 24 paternal chromosomes yielded a maximum lod score of 2.7 (i.e. an increase of 0.6 units) at map position 54.51 cM, corresponding to the rs29027286-rs29021779 interval. The corresponding nominal p-value is 0.0025. Marked drops in the lod score profiles on either side of the peak defined the rs29027283 – rs29013747 interval selected for examination of positional candidates (Additional file 3).

Including LD information from the maternal chromosomes resulted in a decrease of the lod scores in the area corresponding to the linkage peak. Thus the maternal chromosomes did not provide LD information that would independently corroborate the linkage signal, on the contrary (Fig. 5B). One possible explanation for this is that the QTL effect estimated from the segregation of the paternal chromosomes was likely inflated.

The same combined linkage and LD analysis was also applied to the available BTA9 data (i.e. the marker density was not increased), but did not improve either significance or mapping precision (data not shown).

## Discussion and Conlusion

In this work we have first estimated the heritability of gastrointestinal nematode burden in the Dutch Holstein-Friesian dairy cattle population. Parasite load was quantified using faecal egg counts (EPG) in animals of at least two years of age. In a first data set, *h*^2 ^estimates for EPG obtained were relatively high, of the order of 0.20. Stratifying the parasites by species using faecal larval counts did not increase *h*^2^, with the noticeable exception for *Haemonchus *sp. Although *Haemonchus *sp. can infect cattle (e.g., [50]), this high *h*^2 ^is puzzling, because until now evidence for a genetic component of host resistance to this ovine parasite has not been found. In general, herd effects were of the same magnitude as corresponding *h*^2^, including for *Haemonchus *sp larval counts. The significant herd effect in case of *Haemonchus *sp can in part be explained by the fact that this species (*H. contortus*) only occurs in cattle if sheep are present on the farm. In a second data set, however, *h*^2 ^for EPG, estimated using both an animal model and a half-sib design (e.g. [41]), were less than 0.10. This drop may be due to the use of a faster but less sensitive egg counting procedure in this cohort.

We then conducted a whole genome scan to identify QTL affecting faecal egg count. The study was certainly amongst the largest conducted to date as it involved the phenotyping of over 4,000 animals and genotyping of more than 750 within-family extremes. It is worthwhile noting, however, that other QTL mapping studies targeting parasite resistance typically used experimentally challenged animals and/or relied on repeated phenotypic measurements of, for instance, faecal egg counts, which is expected to increase the heritability of the analyzed traits (e.g. [27-29]). The genome scan revealed two chromosome segments with strong evidence for QTL, respectively on BTA9 and BTA19. Although these findings certainly await independent confirmation in cattle, it is noteworthy that in sheep, Crawford et al. [29] detected suggestive QTL influencing faecal egg counts at the orthologous positions on OAR8 (BTA9) and OAR11 (BTA19), while Beh et al. [27] detected a suggestive QTL for faecal egg count at the orthologous OAR11 position. These coincident findings considerably strengthen the support for the detected QTL.

In addition we report six loci with suggestive within-family evidence for QTL affecting faecal egg count. This information might be useful for comparison with results of future studies. Note that because our experimental design was primarily based on linkage analysis in paternal half-sib families, we have not included markers on the X chromosome in this study [51].

We have attempted to refine the map position of the BTA19 QTL using a denser map and by exploiting LD. This effort did not result in substantial gains in terms of signal strength and resolution. This is possibly due to the fact that the marker density was still insufficient to effectively capture an LD signal (Fig. 5A). In particular, this might be the case if the segregating QTL alleles are old and have therefore had ample opportunity to recombine with flanking markers. Alternatively the QTL may be characterized by multiple, individually rare causative alleles that would be more difficult to detect by association mapping.

Genome-wide SNP arrays with a marker density that is at least one order of magnitude higher than what was used in the present study (e.g. [52]) are becoming available in livestock including cattle. It may be worthwhile to genotype the pedigree material used in the present study and attempt to find additional QTL by performing genome-wide association studies. One advantage of association-based designs is that they extract information from the maternal chromosomes whereas the daughter design [32] only uses information from the paternal chromosomes albeit in a very robust manner. LD-based approaches thus have the potential to double the amount of usable information.

The strongest signal for the BTA19 QTL was obtained in an interval flanked by markers rs29027283 and rs29013747. The corresponding chromosome segment measures ~3.3 Mb in the bovine (Btau4.0). Ninety-five genes are annotated to the orthologous genome segment in human (Additional file 3). At least one of these is worth mentioning: *ITGAE*. This gene codes for an integrin alpha chain that is preferentially expressed on the surface of intestinal intraepithelial T lymphocytes, which are thought to be important for immune surveillance and immune responses to mucosal pathogens [53-55].

It is noteworthy that BTA19 harbours several QTL influencing a range of phenotypes of economic importance in dairy cattle, including milk yield and composition and fertility (Coppieters et al., unpublished observations). The impact of selecting for increased parasite resistance on other traits will thus have to be evaluated carefully prior to any MAS attempt.

## Methods

### Determining faecal egg and larval counts

Faeces were collected rectally. Egg flotation and precipitation based on dense salt- and sucrose solutions was used to determine EPG [39]. In brief, for the 2000 cohort 3 – 6 g of faeces was suspended in 25 ml of 13% w/v NaCl solution, followed by filtration over a coarse sieve. After centrifugation, the supernatant was diluted once with tap water, mixed, and precipitated by centrifugation. Subsequently, a flotation and precipitation step in 25 ml of 17% w/v sucrose solution was used to improve clarity of the egg preparations. After removal of the supernatant, digital egg preparations were made as described [39]. For the 2002 cohort, only 2–3 g of faeces was used to be able to analyze larger numbers of faecal samples. Larval cultures to determine larval counts and to identify the involved genera and species were carried out according to [40].

### Variance component analysis

Variance component analyses were conducted with AI-REML [42]. Asymptotic standard errors of the parameters were obtained from the inverse of the Average Information matrix.

### Map construction and information content

Marker maps were constructed using CRIMAP [43]. Linkage information content of the maps was computed as previously described [45]. The information content quantifies the amount of information that is extracted from the marker genotypes as a fraction of the theoretical maximum, i.e. assuming that one could unambiguously determine which paternal homologue was transmitted to each one of the offspring at a given map position.

### QTL mapping

QTL mapping was performed using the rank-based nonparametric option of the previously described HSQM software for QTL mapping in multiple outbred half-sib pedigrees [43]. Briefly, phenotypic values (in this case EPG or BV_LEPG_) are converted to ranks within half-sib pedigrees. Evidence for the segregation of a QTL at a given map position in a given half-sib pedigree, is measured using a test statistic that is closely related to Wilcoxon's sum of rank statistic, yet accounts for the probabilities of inheritance of the grand-maternal versus grand-paternal homologues from the sire. The latter transmission probabilities are computed conditional on marker genotypes. Under the null hypothesis, this pedigree-specific test statistic has a standard normal distribution. Pedigree-specific test statistics are summed over sire families to yield an overall chi-squared statistic with degrees of freedom corresponding to the number of sires. The genome is systematically scanned for the presence using a typical interval mapping procedure, in which the position of the hypothetical QTL slides across the genome at one centimorgan intervals.

The genome-wide significance of this chi-squared statistics (i.e. accounting for the testing of multiple locations across the genome) is determined by comparison with the distribution of the largest genome-wide test statistics obtained from the analysis of 10,000 phenotype permutations [46]. The statistical significance of a QTL was expressed as log(1/*p*), where *p *is the proportion of phenotype permutations for which the QTL test statistic was exceeded anywhere across the genome. QTL were considered significant when log(1/*p*) was superior to 1.30 corresponding to a *p *value of 0.05. Following Lander and Kruglyak [47], QTL were considered suggestive if the test statistic exceeded a threshold reached on average once per phenotype permutation. Assuming that "threshold-exceeding-events" are Poisson distributed, the proportion of permutations for which the suggestive threshold is not exceeded anywhere in the genome is e^-1 ^= 0.37. The *p *value corresponding to the suggestive threshold thus corresponds to the proportion of permutations for which the suggestive threshold is exceeded at least once across the genome which is 1-0.37 = 0.63, and the corresponding log(1/*p*) = 0.2. Confidence intervals for QTL locations were determined by bootstrapping [48]. Bootstrap samples were generated by randomly sampling daughters with replacement, yet keeping the number of daughters constant per family. QTL mapping was conducted on each bootstrap sample using HSQM, and the position with most significant signal stored for each sample. The QTL confidence interval was then defined as the shortest continuous chromosome segment encompassing 95% of these most significant positions.

### Linkage disequilibrium

Pair-wise linkage disequilibrium was quantified using *D' *and *r*^2 ^which were computed from the maternal chromosomes as [56-58].

### QTL fine-mapping

QTL fine-mapping was performed using a previously described variance component approach that simultaneously exploits linkage and linkage disequilibrium [36]. Briefly, we first determined the most likely marker linkage phase for all genotyped animals (i.e. sires and daughters) using purpose-built software (Farnir, unpublished). For a given map position (queried for the location of a QTL), we then (i) computed transmission probabilities for alternate paternal homologues conditional on marker data using standard procedures (e.g. [45]), and (ii) computed identity-by-descent (IBD) probabilities between all pairs of "founder" chromosomes following Meuwissen and Goddard [35]. Founder chromosomes are defined as the 24 chromosomes of the 12 sires as well as the 768 maternal chromosomes of the daughters. Founder chromosomes were clustered on the basis of the pair-wise IBD probabilities using an agglomerative clustering method and the complete link criterion function implemented with CLUTO [36]. The phenotypes of the daughters (LEPG) were first precorrected for fixed effects (herd, parity, days in milk) estimated from the complete data set, and then modelled using a mixed model including a random "founder cluster" effect and a random individual animal effect [41]. Daughters were linked to the clusters of founder chromosomes using coefficients of one (or zero when extracting information from the paternal chromosomes only) for the cluster including the maternal chromosome, coefficients corresponding to the transmission probabilities (or zero when extracting information from the maternal chromosomes only) for the clusters including the respective paternal chromosomes, and coefficients of zero for all other clusters. Corresponding coefficients were summed when two or more parental founder chromosomes belonged to the same cluster. Variance components were estimated by REML [41]. Covariances between chromosome clusters were assumed to be zero, while covariances between individual animal effects were assumed to be proportionate to twice the coefficient of coancestry. The likelihood of the data under this "full" model was compared with the likelihood of the data under a reduced model without cluster effect. Evidence for a QTL at a given map position was expressed as the log_10 _of the corresponding likelihood ratio (LR). The corresponding log_10 _(LR) was computed in the middle of each marker interval to generate a location score profile.

## Abbreviations

QTL: quantitative trait locus; PGE: parasitic gastroenteritis; MHC: major histocompatibilty complex; IFNγ: interferon γ; MAS: marker assisted selection; EPG eggs per gram; LPG: larvae per gram; *h*^2^: narrow-sense heritabilitie; AI-REML: average information restricted maximum likelihood; ANOVA: analysis of variance; BV: breeding value; BTA: *Bos Taurus *autosome; SNP: single nucleotide polymorphism; MAF: minor allele frequency; LD: linkage disequilibrium; IBD: identity-by-descent; LR: log ratio.

## Authors' contributions

WC coordinated microsatellite genotyping, constructed linkage maps, performed QTL and bioinformatic analyses, and participated in writing the manuscript. THMM designed the experiment, collected samples and phenotypes (faecal egg and larval counts) and participated in writing the manuscript. TD & FF estimated heritabilities and performed the combined linkage and LD QTL mapping. NT performed microsatellite genotyping. CS selected the animals to be sampled and compiled genealogical information. AWCAC designed and coordinated the experiment. MG designed and coordinated the experiment, analyzed data and wrote the manuscript. HWP designed the experiment, collected samples and phenotypes (faecal egg and larval counts) and participated in writing the manuscript. All authors read and approved the final manuscript.

## Supplementary Material

Additional file 1 **Frequency distribution of minor allele frequencies for the 73 BTA19 SNPs genotyped for fine-mapping purposes.** Click here for file

Additional file 2 **Combined microsatellite and SNP linkage map of BTA19 used in this study.** Position in cM, number of alleles (N° all), minimum (MinAF), maximum (MaxAF) allele frequency and hterozygosity (Het) for all the markers is listed. Click here for file

Additional file 3 **Genes annotated to the human chromosome segment orthologous to the bovine rs29027283 and rs29013747 interval.** Click here for file

## References

[B1] Gross SJ, Ryan WG, Ploeger HW (1999). Anthelmintic treatment of dairy cows and its effect on milk production. Vet Rec.

[B2] Ketzis JK, Vercruysse J, Stromberg BE, Larsen M, Athanasiadou S, Houdijk JG (2006). Evaluation of efficacy expectations for novel and non-chemical helminth control strategies in ruminants. Vet Parasitol.

[B3] Stear MJ, Doligalska M, Donskow-Schmelter K (2007). Alternatives to anthelmintics for the control of nematodes in livestock. Parasitology.

[B4] Bisset SA, Morris CA, McEwan JC, Vlassoff A (2001). Breeding sheep in New Zealand that are less reliant on anthelmintics to maintain health and productivity. N Z Vet J.

[B5] Windon RG (1990). Selective breeding for the control of nematodiasis in sheep. Rev Sci Tech.

[B6] Woolaston RR, Barger IA, Piper LR (1990). Response to helminth infection of sheep selected for resistance to Haemonchus contortus. Int J Parasitol.

[B7] Baker RL, Watson TG, Bisset SA, Vlassof A, Douch PGC, Gray GD, Woolaston RR (1991). Breeding sheep in New Zealand for resistance to internal parasites: Research results and commercial application. Breeding for Disease Resistance in Sheep.

[B8] Bishop SC, Bairden K, McKellar QA, Park M, Stear MJ (1996). The inheritance of faecal egg count following natural Ostertagia circumcincta infection in Scottish Blackface lambs. Anim Sci.

[B9] Baker RL, Mwamachi DM, Audho JO, Aduda EO, Thorpe W (1999). Genetic resistance to gastro-intestinal nematode parasites in Red Maasai, Dorper and Red Maasai × Dorper ewes in the sub-humid tropics. Anim Sci.

[B10] Gruner L, Aumont G, Getachew T, Brunel JC, Pery C, Cognie Y, Guerin Y (2003). Experimental infection of Black Belly and INRA 401 straight and crossbred sheep with trichostrongyle nematode parasites. Vet Parasitol.

[B11] Nimbkar C, Ghjalsasi PM, Swan AA, Walkden-Brown SW, Kahn LP (2003). Evaluation of growth rates and resistance to nematodes of Deccani and Bannur lambs and their crosses with Garole. Anim Sci.

[B12] Morris CA, Vlassoff A, Bisset SA, Baker RL, West CJ, Hurford AP (1997). Responses of Romney sheep to selection for resistance or susceptibility to nematode infection. Anim Sci.

[B13] Gasbarre LC, Sonstegaard T, Van Tassell CP, Padilha T Detection of QTL affecting parasite resistance in a selected herd of Angus cattle. Proceedings of the 7th World Congress on Genetics Applied to Livestock Production: 19–23 August 2002 Montpellier, France.

[B14] Bisset SA, Vlassoff A, Morris CA, Southey BR, Baker RL, Parker AGH (1992). Heritability and genetic correlations among faecal egg counts and productivity traits in Romney sheep. New Zealand J Agric Res.

[B15] Eady SJ, Woolaston RR, Lewer RP, Raadsma HW, Swan AA, Ponzoni R (1996). Resistance to nematode parasites in Merino sheep: correlation with production traits. Aust J Agric Res.

[B16] Bouix J, Krupinski J, Rzepecki R, Nowosad B, Skrzyzala I, Roborzynski M, Fudalewicz-Niemczyk W, Skalska M, Malczewski A, Gruner L (1998). Genetic resistance to gastrointestinal nematode parasites in Polish long-wool sheep. International Journal of Parasitology.

[B17] Woolaston RR, Windon RG (2001). Selection of sheep for response to Trichostrongylus colubriformis larvae: genetic parameters. Anim Sci.

[B18] Vagenas D, Jackson F, Russel AJF, Merchant M, Wright IA, Bishop SC (2002). Genetic control of resistance to gastro-intestinal parasites in crossbred cashmere-producing goats: responses to selection, genetic parameters and relationships with production traits. Anim Sci.

[B19] Morris CA, Green RS, Cullen NG, Hickey SM (2003). Genetic and phenotypic relationships among faecal egg count, anti-nematode antibody level and live weight in Angus cattle. Anim Sci.

[B20] Morris CA, Green RS, Hickey SM, Auldist MJ, Thomson NA, Cullen NG (2004). Relationships among faecal egg counts, anti-parasite antibodies and milk yields in an experimental Friesian herd. New Zealand J Agric Res.

[B21] Morris CA, Wheeler M, Watson TG, Hosking BC, Leathwick DM (2005). Direct and correlated responses to selection for high or low faecal nematode egg count in Perendale sheep. New Zealand J Agric Res.

[B22] Gasbarre LC, Leighton EA, Sonstegard T (2001). Role of the bovine immune system and genome in resistance to gastrointestinal nematodes. Vet Parasitol.

[B23] Dominik S (2005). Quantitative Trait Loci for internal nematode resistance in sheep: a review. Genet Sel Evol.

[B24] Stear MJ, Bairden K, Bishop SC, Buitkamp J, Epplen JT, Gostomski D, McKellar QA, Schwaiger FW, Wallace DS (1996). An ovine lymphocyte antigen is associated with reduced faecal egg counts in four-month-old lambs following natural, predominantly Ostertagia circumcincta infection. Int J Parasitol.

[B25] Paterson S, Wilson K, Pemberton JM (1998). Major histocompatibility complex variation associated with juvenile survival and parasite resistance in a large unmanaged ungulate population (Ovis aries L.). Proc Natl Acad Sci USA.

[B26] Coltman DW, Wilson K, Pilkington JG, Stear MJ, Pemberton JM (2001). A microsatellite polymorphism in the gamma interferon gene is associated with resistance to gastrointestinal nematodes in a naturally-parasitized population of Soay sheep. Parasitology.

[B27] Beh KJ, Hulme DJ, Callaghan MJ, Leish Z, Lenane I, Windon RG, Maddox JF (2002). A genome scan for quantitative trait loci affecting resistance to Trichostrongylus colubriformis in sheep. Animal Genetics.

[B28] Davies G, Stear MJ, Benothman M, Abuagob O, Kerr A, Mitchell S, Bishop SC (2006). Quantitative trait loci associated with parasitic infection in Scottish blackface sheep. Heredity.

[B29] Crawford AM, Paterson KA, Dodds KG, Diez-Tascon C, Williamson PA, Roberts Thomsom M, Bisset SA, Beattie AE, Greer GJ, Green RS, Wheeler R, Shaw RJ, Knowler K, McEwan JC (2006). Discovery of quantitative trait loci for resistance to parasitic nematode infection in sheep: I. Analysis of outcross pedigrees. BMC Genomics.

[B30] Behnke JM, Iraqi FA, Mugambi JM, Clifford S, Nagda S, Wakelin D, Kemp SJ, Baker RL, Gibson JP (2006). High resolution mapping of chromosomal regions controlling resistance to gastrointestinal nematode infections in an advanced intercross line of mice. Mamm Genome.

[B31] Williams-Blangero S, VandeBerg JL, Subedi J, Aivaliotis MJ, Rai DR, Upadhayay RP, Jha B, Blangero J (2002). Genes on chromosomes 1 and 13 have significant effects on Ascaris infection. Proc Natl Acad Sci USA.

[B32] Weller JI, Kashi Y, Soller M (1990). Power of daughter and granddaughter designs for determining linkage between marker loci and quantitative trait loci in dairy cattle. J Dairy Sci.

[B33] Lander ES, Botstein D (1989). Mapping mendelian factors underlying quantitative traits using RFLP linkage maps. Genetics.

[B34] Meuwissen TH, Goddard ME (2000). Fine mapping of quantitative trait loci using linkage disequilibria with closely linked marker loci. Genetics.

[B35] Meuwissen TH, Goddard ME (2001). Prediction of identity by descent probabilities from marker-haplotypes. Genet Sel Evol.

[B36] Blott S, Kim JJ, Moisio S, Schmidt-Kuntzel A, Cornet A, Berzi P, Cambisano N, Ford C, Grisart B, Johnson D, Karim L, Simon P, Snell R, Spelman R, Wong J, Vilkki J, Georges M, Farnir F, Coppieters W (2003). Molecular dissection of a quantitative trait locus: a phenylalanine-to-tyrosine substitution in the transmembrane domain of the bovine growth hormone receptor is associated with a major effect on milk yield and composition. Genetics.

[B37] Eysker M, Van Meurs GK (1982). Seasonal pattern in the strongyle egg output of adult dairy cows in the Netherlands. Res Vet Sci.

[B38] Kloosterman A (1983). Toepassingen van de immunologie in de veterinaire helminthologie. Tijdschrift voor Diergeneeskunde.

[B39] Mes TH, Ploeger HW, Terlou M, Kooyman FN, Ploeg MP van der, Eysker M (2001). A novel method for the isolation of gastro-intestinal nematode eggs that allows automated analysis of digital images of egg preparations and high throughput screening. Parasitology.

[B40] Borgsteede FHM, Hendriks J (1974). Identification of infective larvae of gastro-intestinal nematodes in cattle. Tijdschr Diergeneeskunde.

[B41] Lynch M, Walsh B (1997). Genetics and analysis of quantitative traits.

[B42] Johnson DL, Thompson R (1995). Restricted maximum likelihood estimation of variance components for univariate Animal Models using sparse matrix techniques and average information. J Dairy Sci.

[B43] Lander ES, Green P (1987). Construction of Multilocus Genetic Linkage Maps in Humans. Proc Natl Acad Sci USA.

[B44] Ihara N, Takasuga A, Mizoshita K, Takeda H, Sugimoto M, Mizoguchi Y, Hirano T, Itoh T, Watanabe T, Reed KM, Snelling WM, Kappes SM, Beattie CW, Bennett GL, Sugimoto Y (2004). A comprehensive genetic map of the cattle genome based on 3802 microsatellites. Genome Res.

[B45] Coppieters W, Kvasz A, Farnir F, Arranz JJ, Grisart B, Mackinnon M, Georges M (1998). A rank-based nonparametric method for mapping quantitative trait loci in outbred half-sib pedigrees: application to milk production in a granddaughter design. Genetics.

[B46] Doerge RW, Churchill GA (1996). Permutation tests for multiple loci affecting a quantitative character. Genetics.

[B47] Lander ES, Kruglyak L (1995). Genetic dissection of complex traits, guidelines for interpreting and reporting linkage results. Nat Genet.

[B48] Visscher PM, Thompson R, Haley CS (1996). Confidence intervals in QTL mapping by bootstrapping. Genetics.

[B49] Beavis WD, Patterson AH (1998). QTL analysis: power, precision and accuracy. Molecular Dissection of Complex Traits.

[B50] Amarante AFT, Bagnola J, Amarante MRV, Barbosa MA (1997). Host specificity of sheep and cattle nematodes in Sao Paolo state, Brazil. Vet Parasitol.

[B51] Sandor C, Farnir F, Hansoul S, Coppieters W, Meuwissen T, Georges M (2006). Linkage disequilibrium on the bovine X chromosome: characterization and use in quantitative trait locus mapping. Genetic.

[B52] Charlier C, Coppieters W, Rollin F, Desmecht D, Agerholm JS, Cambisano N, Carta E, Dardano S, Dive M, Fasquelle C, Frennet JC, Hanset R, Hubin X, Jorgensen C, Karim L, Kent M, Harvey K, Pearce BR, Simon P, Tama N, Nie H, Vandeputte S, Lien S, Longeri M, Fredholm M, Harvey RJ, Georges M (2008). Highly effective SNP-based association mapping and management of recessive defects in livestock. Nat Genet.

[B53] Cerf-Bensussan N, Jarry A, Brousse N, Lisowska-Grospierre B, Guy-Grand D, Griscelli C (1987). A monoclonal antibody (HML-1) defining a novel membrane molecule present on human intestinal lymphocytes. Eur J Immunol.

[B54] Schon MP, Arya A, Murphy EA, Adams CM, Strauch UG, Agace WW, Marsal J, Donohue JP, Her H, Beier DR, Olson S, Lefrancois L, Brenner MB, Grusby MJ, Parker CM (1999). Mucosal T lymphocyte numbers are selectively reduced in integrin alpha E (CD103)-deficient mice. J Immunol.

[B55] Parker CM, Cepek KL, Russell GJ, Shaw SK, Posnett DN, Schwarting R, Brenner MB (1992). A family of beta 7 integrins on human mucosal lymphocytes. Proc Natl Acad Sci USA.

[B56] Hedrick PW (1987). Gametic disequilibrium measures: proceed with caution. Genetics.

[B57] Farnir F, Coppieters W, Arranz J-J, Berzi P, Cambisano N, Grisart B, Karim L, Marcq F, Moreau L, Mni M, Nezer C, Simon P, Vanmanshoven P, Wagenaar D, Georges M (2000). Extensive genome-wide linkage disequilibrium in cattle. Genome Research.

[B58] Grisart B, Farnir F, Karim L, Cambisano N, Kim J-J, Kvasz A, Mni M, Simon P, Frere J-M, Coppieters W, Georges M (2004). Genetic and functional demonstration of the causality of the DGAT1 K232A mutation in the determinism of the BTA14 QTL affecting milk yield and composition. Natl Acad Sci USA.

