# Supplementary figures and images for: Mapping QTL influencing gastrointestinal nematode burden in Dutch Holstein-Friesian dairy cattle
Source: BMC Genomics. 2009 Mar 2;10:96. doi: 10.1186/1471-2164-10-96 (PMC2657155; doi:10.1186/1471-2164-10-96)

## Slide 1
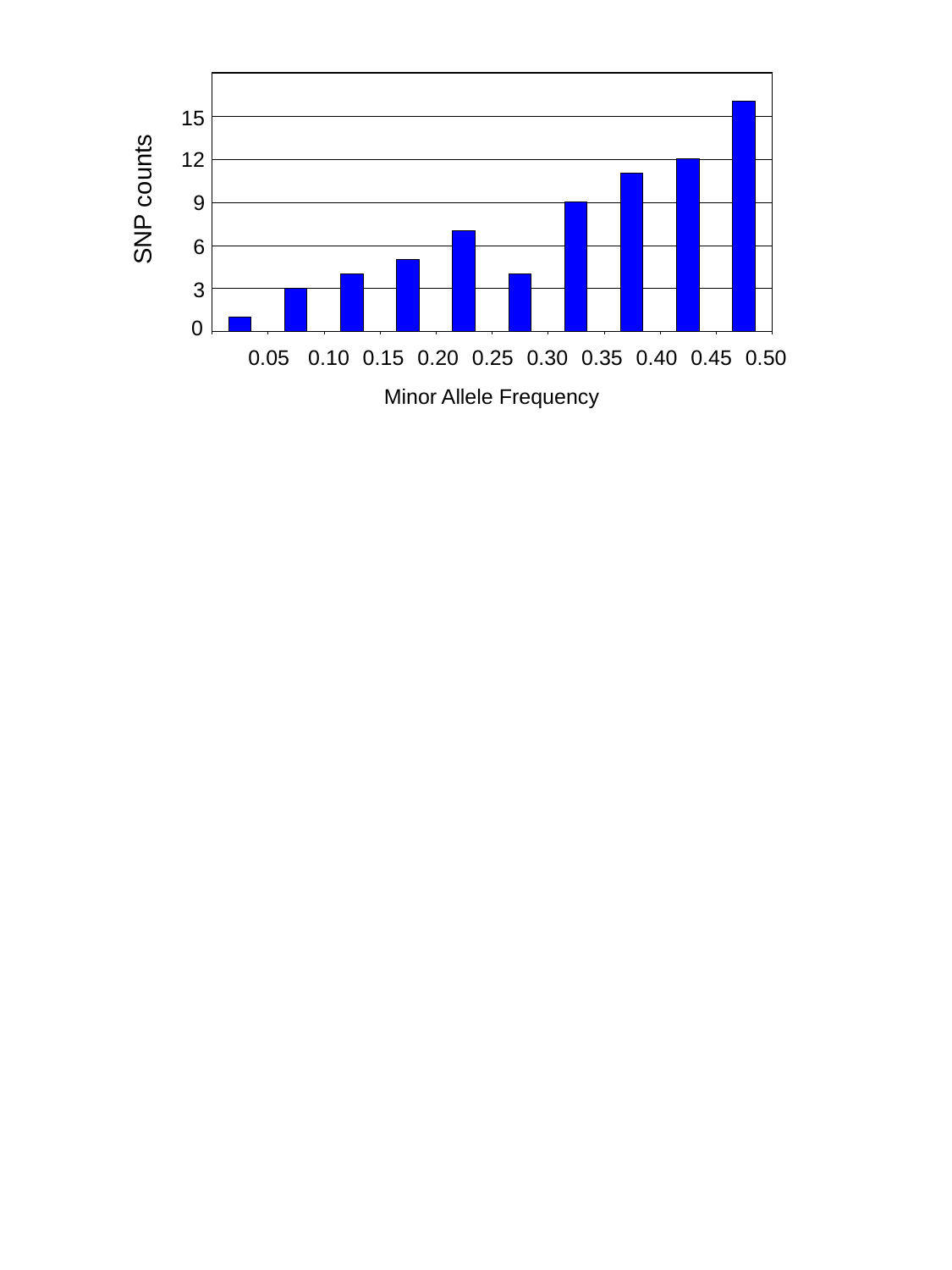

15
12
SNP counts
 9
 6
 3
 0
0.05
0.10
0.15
0.20
0.25
0.30
0.35
0.40
0.45
0.50
Minor Allele Frequency

Supplement: Additional file 1 — Frequency distribution of minor allele frequencies for the 73 BTA19 SNPs genotyped for fine-mapping purposes. [file 1471-2164-10-96-S1.ppt]
