# Supplementary material for: Mapping QTL influencing gastrointestinal nematode burden in Dutch Holstein-Friesian dairy cattle
Source: BMC Genomics. 2009 Mar 2;10:96. doi: 10.1186/1471-2164-10-96 (PMC2657155; doi:10.1186/1471-2164-10-96)
Supplement: Additional file 2 — Combined microsatellite and SNP linkage map of BTA19 used in this study. Position in cM, number of alleles (N° all), minimum (MinAF), maximum (MaxAF) allele frequency and hterozygosity (Het) for all the markers is listed. [file 1471-2164-10-96-S2.doc]

| N° | Marker | Pos (cM) | Het | n° all | MinAF | MaxAF |
| --- | --- | --- | --- | --- | --- | --- |
| 1 | BM9202_1:1 | 0,00 | 0,77 | 10 | 0,00 | 0,32 |
| 2 | rs29018587 | 3,00 | 0,43 | 2 | 0,32 | 0,68 |
| 3 | rs29014584 | 3,00 | 0,25 | 2 | 0,15 | 0,85 |
| 4 | rs29027227 | 3,00 | 0,45 | 2 | 0,35 | 0,65 |
| 5 | rs29014586 | 5,00 | 0,47 | 2 | 0,37 | 0,63 |
| 6 | rs29027040 | 14,10 | 0,46 | 2 | 0,37 | 0,63 |
| 7 | rs29021246 | 14,10 | 0,31 | 2 | 0,19 | 0,81 |
| 8 | rs29027039 | 16,10 | 0,28 | 2 | 0,17 | 0,83 |
| 9 | rs29021247 | 16,10 | 0,30 | 2 | 0,19 | 0,81 |
| 10 | rs29021248 | 19,11 | 0,30 | 2 | 0,18 | 0,82 |
| 11 | HEL10_1:1 | 19,11 | 0,62 | 7 | 0,00 | 0,47 |
| 12 | rs29018185 | 20,11 | 0,49 | 2 | 0,43 | 0,57 |
| 13 | BMS745_1:1 | 21,11 | 0,73 | 10 | 0,00 | 0,45 |
| 14 | rs29011454 | 22,11 | 0,09 | 2 | 0,05 | 0,95 |
| 15 | DIK1124_1:1 | 22,11 | 0,28 | 2 | 0,17 | 0,83 |
| 16 | rs29013474 | 22,11 | 0,34 | 2 | 0,21 | 0,79 |
| 17 | AFL361_1:1 | 23,11 | 0,22 | 7 | 0,01 | 0,88 |
| 18 | TGLA94_1:1 | 24,11 | 0,60 | 7 | 0,00 | 0,57 |
| 19 | rs29025380 | 24,11 | 0,48 | 2 | 0,40 | 0,60 |
| 20 | rs29015001 | 25,11 | 0,14 | 2 | 0,07 | 0,93 |
| 21 | rs29022102 | 26,11 | 0,50 | 2 | 0,48 | 0,52 |
| 22 | rs29027469 | 26,11 | 0,25 | 2 | 0,15 | 0,85 |
| 23 | rs29020856 | 30,12 | 0,17 | 2 | 0,10 | 0,90 |
| 24 | rs29027471 | 30,12 | 0,25 | 2 | 0,14 | 0,86 |
| 25 | BMC5012_1:1 | 30,12 | 0,55 | 4 | 0,03 | 0,59 |
| 26 | DIK2067_1:1 | 31,12 | 0,09 | 3 | 0,00 | 0,95 |
| 27 | INRABERN148_1:1 | 34,12 | 0,78 | 11 | 0,00 | 0,27 |
| 28 | rs29017666 | 36,12 | 0,49 | 2 | 0,42 | 0,58 |
| 29 | rs29026496 | 36,12 | 0,41 | 2 | 0,29 | 0,71 |
| 30 | rs29018296 | 40,13 | 0,49 | 2 | 0,42 | 0,58 |
| 31 | rs29012309 | 45,15 | 0,46 | 2 | 0,36 | 0,64 |
| 32 | rs29022376 | 45,15 | 0,50 | 2 | 0,47 | 0,53 |
| 33 | rs29020857 | 49,16 | 0,32 | 2 | 0,20 | 0,80 |
| 34 | rs29027283 | 49,16 | 0,48 | 2 | 0,41 | 0,59 |
| 35 | rs29021777 | 52,16 | 0,40 | 2 | 0,28 | 0,72 |
| 36 | rs29012543 | 52,16 | 0,50 | 2 | 0,49 | 0,51 |
| 37 | rs29012538 | 52,16 | 0,49 | 2 | 0,43 | 0,57 |
| 38 | rs29012541 | 52,16 | 0,49 | 2 | 0,43 | 0,57 |
| 39 | BMS1920_1:1 | 52,16 | 0,74 | 7 | 0,00 | 0,31 |
| 40 | URB046_1:1 | 52,16 | 0,56 | 4 | 0,01 | 0,54 |
| 41 | URB026_1:1 | 52,16 | 0,55 | 4 | 0,01 | 0,55 |
| 42 | TGLA51_1:1 | 52,16 | 0,68 | 5 | 0,01 | 0,48 |
| 43 | rs29027492 | 52,16 | 0,50 | 2 | 0,49 | 0,51 |
| 44 | DIK2070_1:1 | 52,16 | 0,69 | 7 | 0,00 | 0,44 |
| 45 | rs29027286 | 52,16 | 0,50 | 2 | 0,46 | 0,54 |
| 46 | rs29021779 | 52,16 | 0,48 | 2 | 0,39 | 0,61 |
| 47 | rs29012542 | 53,16 | 0,44 | 2 | 0,32 | 0,68 |
| 48 | RM222_1:1 | 54,16 | 0,72 | 7 | 0,00 | 0,41 |
| 49 | rs29027062 | 55,16 | 0,43 | 2 | 0,31 | 0,69 |
| 50 | rs29013747 | 55,16 | 0,50 | 2 | 0,49 | 0,51 |
| 51 | TEXAN12_1:1 | 56,16 | 0,51 | 4 | 0,01 | 0,66 |
| 52 | rs29022944 | 56,16 | 0,40 | 2 | 0,28 | 0,72 |
| 53 | rs29027074 | 57,16 | 0,44 | 2 | 0,33 | 0,67 |
| 54 | CSSME070_1:1 | 58,16 | 0,59 | 4 | 0,01 | 0,55 |
| 55 | BMS2142_1:1 | 62,17 | 0,79 | 11 | 0,01 | 0,26 |
| 56 | rs29018088 | 63,17 | 0,46 | 2 | 0,37 | 0,63 |
| 57 | rs29022451 | 64,17 | 0,45 | 2 | 0,34 | 0,66 |
| 58 | rs29020054 | 64,17 | 0,48 | 2 | 0,39 | 0,61 |
| 59 | ILSTS014_1:1 | 65,17 | 0,46 | 3 | 0,00 | 0,64 |
| 60 | rs29010293 | 65,17 | 0,49 | 2 | 0,43 | 0,57 |
| 61 | rs29018893 | 66,17 | 0,44 | 2 | 0,33 | 0,67 |
| 62 | rs29022537 | 68,17 | 0,35 | 2 | 0,22 | 0,78 |
| 63 | rs29017013 | 68,17 | 0,50 | 2 | 0,46 | 0,54 |
| 64 | UWCA40_1:1 | 69,17 | 0,31 | 3 | 0,01 | 0,81 |
| 65 | rs29021711 | 77,24 | 0,41 | 2 | 0,28 | 0,72 |
| 66 | rs29022544 | 77,24 | 0,50 | 2 | 0,47 | 0,53 |
| 67 | rs29015945 | 79,24 | 0,50 | 2 | 0,49 | 0,51 |
| 68 | DIK721_1:1 | 80,24 | 0,16 | 4 | 0,01 | 0,91 |
| 69 | BM17132_1:1 | 80,24 | 0,77 | 10 | 0,00 | 0,28 |
| 70 | rs29025958 | 81,24 | 0,49 | 2 | 0,45 | 0,55 |
| 71 | DIK2486_1:1 | 81,24 | 0,58 | 5 | 0,01 | 0,54 |
| 72 | rs29017737 | 81,24 | 0,24 | 2 | 0,14 | 0,86 |
| 73 | KRT10_1:1 | 82,24 | 0,55 | 3 | 0,15 | 0,61 |
| 74 | rs29015057 | 84,24 | 0,47 | 2 | 0,37 | 0,63 |
| 75 | rs29015754 | 86,24 | 0,37 | 2 | 0,24 | 0,76 |
| 76 | rs29021794 | 89,25 | 0,49 | 2 | 0,44 | 0,56 |
| 77 | BMS501_1:1 | 89,25 | 0,59 | 7 | 0,00 | 0,57 |
| 78 | rs29021793 | 97,32 | 0,50 | 2 | 0,48 | 0,52 |
| 79 | BL1006_1:1 | 98,32 | 0,58 | 4 | 0,06 | 0,59 |
| 80 | rs29017035 | 102,33 | 0,45 | 2 | 0,34 | 0,66 |
| 81 | rs29013561 | 106,33 | 0,50 | 2 | 0,48 | 0,52 |
| 82 | rs29013562 | 106,33 | 0,15 | 2 | 0,08 | 0,92 |
| 83 | rs29019828 | 106,33 | 0,36 | 2 | 0,23 | 0,77 |
| 84 | rs29010396 | 106,33 | 0,46 | 2 | 0,35 | 0,65 |
| 85 | rs29017034 | 112,36 | 0,46 | 2 | 0,35 | 0,65 |
| 86 | rs29011140 | 112,36 | 0,50 | 2 | 0,49 | 0,51 |
| 87 | rs29011141 | 113,36 | 0,47 | 2 | 0,38 | 0,62 |
| 88 | IDVGA44_1:1 | 115,36 | 0,63 | 9 | 0,00 | 0,56 |
| 89 | rs29017164 | 116,36 | 0,45 | 2 | 0,34 | 0,66 |
| 90 | NLBCMK25_1:1 | 123,41 | 0,85 | 10 | 0,01 | 0,26 |
| 91 | rs29020026 | 124,41 | 0,49 | 2 | 0,42 | 0,58 |
| 92 | rs29023185 | 125,41 | 0,37 | 2 | 0,24 | 0,76 |
| 93 | rs29020029 | 125,41 | 0,32 | 2 | 0,20 | 0,80 |
| 94 | rs29020027 | 135,55 | 0,50 | 2 | 0,49 | 0,51 |
| 95 | rs29023182 | 140,56 | 0,48 | 2 | 0,40 | 0,60 |
| 96 | rs29011071 | 146,59 | 0,36 | 2 | 0,23 | 0,77 |
| 97 | BMC1013_1:1 | 146,59 | 0,24 | 4 | 0,01 | 0,87 |
| 98 | rs29027098 | 146,59 | 0,50 | 2 | 0,49 | 0,51 |
| 99 | rs29027099 | 146,59 | 0,49 | 2 | 0,44 | 0,56 |
| 100 | rs29020141 | 151,61 | 0,50 | 2 | 0,47 | 0,53 |
| 101 | rs29027102 | 167,09 | 0,50 | 2 | 0,47 | 0,53 |
