# Supplementary material for: Mapping QTL influencing gastrointestinal nematode burden in Dutch Holstein-Friesian dairy cattle
Source: BMC Genomics. 2009 Mar 2;10:96. doi: 10.1186/1471-2164-10-96 (PMC2657155; doi:10.1186/1471-2164-10-96)
Supplement: Additional file 3 — Genes annotated to the human chromosome segment orthologous to the bovine rs29027283 and rs29013747 interval. [file 1471-2164-10-96-S3.doc]

| Gene | Description |
| --- | --- |
| ENSG00000187939 | No Description |
| RPH3AL,ENSG00000181031 | rabphilin 3A-like (without C2 domains) Generated via Q9UNE2 |
| ENSG00000183518 | No Description |
| NP_001013694.3,ENSG00000187624 | hypothetical protein LOC400566 Relationship generated from exonerate mapping |
| FAM101B,ENSG00000183688 | family with sequence similarity 101, member B Generated via NP_874364 |
| VPS53,ENSG00000141252 | vacuolar protein sorting 53 homolog (S. cerevisiae) Generated via NP_060759 |
| FAM57A,ENSG00000167695 | family with sequence similarity 57, member A Generated via NP_079068 |
| GEMIN4,ENSG00000179409 | gem (nuclear organelle) associated protein 4 Generated via NP_056536 |
| Q9NRE4_HUMAN,ENSG00000179393 | Endozepine-like protein type 2 mutant. Relationship generated from exonerate mapping |
| ENSG00000214061 | No Description |
| GLOD4,ENSG00000167699 | glyoxalase domain containing 4 Generated via NP_057164 |
| ENSG00000196737 | No Description |
| RNMTL1,ENSG00000171861 | RNA methyltransferase like 1 Generated via Q53GN1 |
| NXN,ENSG00000167693 | nucleoredoxin Generated via Q7L4C6 |
| Q6ZP06_HUMAN,ENSG00000188804 | CDNA FLJ26768 fis, clone PRS02994. Relationship generated from exonerate mapping |
| TIMM22,ENSG00000177370 | translocase of inner mitochondrial membrane 22 homolog (yeast) Generated via Q9Y584 |
| ABR,ENSG00000159842 | active BCR-related gene Generated via Q12979 |
| Q7RTU4_HUMAN,ENSG00000205899 | Hypothetical class II basic helix-loop-helix protein (Fragment). Relationship generated from exonerate mapping |
| TUSC5,ENSG00000184811 | tumor suppressor candidate 5 Generated via Q8IXB3 |
| ENSG00000209456 | No Description |
| YWHAE,ENSG00000108953 | tyrosine 3-monooxygenase/tryptophan 5-monooxygenase activation protein, epsilon polypeptide Generated via P62258 |
| CRK,ENSG00000167193 | v-crk sarcoma virus CT10 oncogene homolog (avian) Generated via P46108 |
| MYO1C,ENSG00000197879 | myosin IC Generated via NP_001074419 |
| SKIP_HUMAN,ENSG00000132376 | Skeletal muscle and kidney-enriched inositol phosphatase (EC 3.1.3.56). Relationship generated from exonerate mapping |
| PITPNA,ENSG00000174238 | phosphatidylinositol transfer protein, alpha Generated via Q00169 |
| SLC43A2,ENSG00000167703 | solute carrier family 43, member 2 Generated via NP_689559 |
| ENSG00000209459 | No Description |
| ENSG00000209464 | No Description |
| SCARF1,ENSG00000074660 | scavenger receptor class F, member 1 Generated via NP_003684 |
| RILP,ENSG00000167705 | Rab interacting lysosomal protein Generated via NP_113618 |
| PRPF8,ENSG00000174231 | PRP8 pre-mRNA processing factor 8 homolog (S. cerevisiae) Generated via Q6P2Q9 |
| ENSG00000185561 | No Description |
| NP_116284.2,ENSG00000186594 | hypothetical protein LOC84981 Relationship generated from exonerate mapping |
| hsa-mir-22,ENSG00000199060 | hsa-mir-22 Relationship generated from exonerate mapping |
| WDR81,ENSG00000167716 | WD repeat domain 81 Generated via NP_689561 |
| SERPINF2,ENSG00000167711 | serpin peptidase inhibitor, clade F (alpha-2 antiplasmin, pigment epithelium derived factor), member 2 Generated via NP_000925 |
| ENSG00000205843 | No Description |
| Q6ZV47_HUMAN,ENSG00000205841 | CDNA FLJ42997 fis, clone BRTHA2011351. Relationship generated from exonerate mapping |
| SERPINF1,ENSG00000132386 | serpin peptidase inhibitor, clade F (alpha-2 antiplasmin, pigment epithelium derived factor), member 1 Generated via P36955 |
| SMYD4,ENSG00000186532 | SET and MYND domain containing 4 Generated via NP_443160 |
| RPA1,ENSG00000132383 | replication protein A1, 70kDa Generated via NP_002936 |
| Q3C259_HUMAN,ENSG00000108958 | Succinate dehydrogenase complex, subunit C delta3+5 alternative splicing variant. Relationship generated from exonerate mapping |
| Q7Z4G2_HUMAN,ENSG00000212735 | MSTP075. Relationship generated from exonerate mapping |
| RTN4RL1,ENSG00000185924 | reticulon 4 receptor-like 1 Generated via Q86UN2 |
| DPH1,ENSG00000108963 | DPH1 homolog (S. cerevisiae) Generated via Q9BZG8 |
| NP_543012.1,ENSG00000214014 | candidate tumor suppressor in ovarian cancer 2 Relationship generated from exonerate mapping |
| hsa-mir-132,ENSG00000207724 | hsa-mir-132 Relationship generated from exonerate mapping |
| hsa-mir-212,ENSG00000207953 | hsa-mir-212 Relationship generated from exonerate mapping |
| HIC1,ENSG00000177374 | hypermethylated in cancer 1 Generated via Q14526 |
| SMG6,ENSG00000070366 | Smg-6 homolog, nonsense mediated mRNA decay factor (C. elegans) Generated via Q86US8 |
| ENSG00000215230 | No Description |
| ENSG00000212375 | No Description |
| SRR,ENSG00000167720 | serine racemase Generated via NP_068766 |
| TSR1,ENSG00000167721 | TSR1, 20S rRNA accumulation, homolog (S. cerevisiae) Generated via NP_060598 |
| SNORD91B,ENSG00000212552 | small nucleolar RNA, C/D box 91B Generated via NR_003073 |
| O43147-2,ENSG00000141258 | RUTB1_HUMAN Isoform 2 of O43147 - Homo sapiens (Human) Relationship generated from exonerate mapping |
| MNT,ENSG00000070444 | MAX binding protein Generated via NP_064706 |
| NP_001020630.1,ENSG00000205821 | hypothetical protein LOC284009 Relationship generated from exonerate mapping |
| METT10D,ENSG00000127804 | methyltransferase 10 domain containing Generated via NP_076991 |
| ENSG00000209488 | No Description |
| ENSG00000209492 | No Description |
| PAFAH1B1,ENSG00000007168 | platelet-activating factor acetylhydrolase, isoform Ib, alpha subunit 45kDa Generated via NP_000421 |
| ENSG00000209044 | No Description |
| ENSG00000209099 | No Description |
| KIAA0664,ENSG00000132361 | KIAA0664 Generated via NP_056044 |
| GARNL4,ENSG00000132359 | GTPase activating Rap/RanGAP domain-like 4 Generated via Q684P5 |
| ENSG00000203592 | No Description |
| OR1D5,ENSG00000182880 | olfactory receptor, family 1, subfamily D, member 5 Generated via P58170 |
| OR1D2,ENSG00000184166 | olfactory receptor, family 1, subfamily D, member 2 Generated via P34982 |
| ENSG00000142163 | No Description |
| OR1G1,ENSG00000183024 | olfactory receptor, family 1, subfamily G, member 1 Generated via NP_003546 |
| Q8NH06_HUMAN,ENSG00000180144 | Seven transmembrane helix receptor. Relationship generated from exonerate mapping |
| OR1A2,ENSG00000172150 | olfactory receptor, family 1, subfamily A, member 2 Generated via Q9Y585 |
| OR1A1,ENSG00000172146 | olfactory receptor, family 1, subfamily A, member 1 Generated via NP_055380 |
| OR3A2,ENSG00000205813 | olfactory receptor, family 3, subfamily A, member 2 Generated via NP_002542 |
| OR3A1,ENSG00000180090 | olfactory receptor, family 3, subfamily A, member 1 Generated via P47881 |
| OR3A4,ENSG00000180068 | olfactory receptor, family 3, subfamily A, member 4 Generated via NP_001005334 |
| ENSG00000180042 | No Description |
| OR1E1,ENSG00000180016 | olfactory receptor, family 1, subfamily E, member 1 Generated via P30953 |
| OR3A3,ENSG00000159961 | olfactory receptor, family 3, subfamily A, member 3 Generated via NP_036505 |
| OR1E2,ENSG00000127780 | olfactory receptor, family 1, subfamily E, member 2 Generated via NP_003545 |
| SPATA22,ENSG00000141255 | spermatogenesis associated 22 Generated via NP_115987 |
| ASPA,ENSG00000108381 | aspartoacylase (Canavan disease) Generated via NP_000040 |
| TRPV3,ENSG00000167723 | transient receptor potential cation channel, subfamily V, member 3 Generated via NP_659505 |
| CARKL,ENSG00000197417 | carbohydrate kinase-like Generated via Q9UHJ6 |
| CTNS,ENSG00000040531 | cystinosis, nephropathic Generated via NP_001026851 |
| TAX1BP3,ENSG00000213977 | Tax1 (human T-cell leukemia virus type I) binding protein 3 Generated via NP_055419 |
| TMEM93,ENSG00000127774 | transmembrane protein 93 Generated via Q9BV81 |
| P2RX5,ENSG00000083454 | purinergic receptor P2X, ligand-gated ion channel, 5 Generated via NP_002552 |
| ITGAE,ENSG00000083457 | integrin, alpha E (antigen CD103, human mucosal lymphocyte antigen 1| alpha polypeptide) Generated via P38570 |
| GSG2,ENSG00000177602 | germ cell associated 2 (haspin) Generated via NP_114171 |
| C17orf85,ENSG00000074356 | chromosome 17 open reading frame 85 Generated via NP_061023 |
| CAMKK1,ENSG00000004660 | calcium/calmodulin-dependent protein kinase kinase 1, alpha Generated via Q8N5S9 |
| P2RX1,ENSG00000108405 | purinergic receptor P2X, ligand-gated ion channel, 1 Generated via P51575 |
| ATP2A3,ENSG00000074370 | ATPase, Ca++ transporting, ubiquitous Generated via Q93084 |
